# Causes & Consequences of Health Care CEO Turnover in Australia and Retention Strategies: A Qualitative Study

**DOI:** 10.1177/00469580241233250

**Published:** 2024-02-20

**Authors:** Nebu Varughese Mathew, Chaojie Liu, Hanan Khalil

**Affiliations:** 1 La Trobe University, Melbourne, VIC, Australia

**Keywords:** CEO, turnover, health, causes, consequences, retention

## Abstract

It has been highlighted the increasing CEO turnover is a major issue for Australian and international health care organizations. Some of the negative consequences of CEO turnover includes organizational instability, high financial costs, and affecting patient care. On average, CEOs leave their role within 1 to 2 years after commencement, which can be detrimental to the hospital’s overall functioning. Therefore, this study aims to identify the causes and consequences of increasing CEO turnover in health care, so retention strategies could be devised. Fourteen hospital CEOs across Australia were interviewed online to answer 5 open ended questions related to qualities of a CEO, challenges of the CEO role, Causes and consequences of increasing CEO turnover and recommendation for CEOs retention. Interviews followed a semi-structured schedule to prompt discussion in relation to the above. The study has identified that CEOs possess certain qualities such as self-awareness, leadership style, resilience that enable them to perform their role well. Challenges of this role such as COVID-19, managing organizational change staff management has been found and discussed. Broadly, causes have been divided into 5 major categories such as Organization, Performance, Pressure, Personal and Health reform. It was found that increasing CEO turnover can be damaging to the hospital, not just the staff and patients suffer but the surrounding community gets adversely affected. To minimize CEO turnover, it was suggested that the board needs to support their CEO by advocating and investing in organizational culture and leadership programs. The findings of this study aid, the board with certain strategies through which CEO can be supported. CEOs made certain recommendations in this study to minimize the turnover which can make such a big impact on health care as this may lead to better functioning hospitals in Australia. Moreover, these strategies could be used internationally to help them CEOs retain in their position.

**What do we already know about this topic**
There has been some research done in relation to health executive turnover in Australia. Consequences of increasing health executive turnover has been explored. Some causes of increasing turnover have been discussed.
**How does your research contribute to the field?**
This is the only study that has explored the causes of CEO turnover in Australia. This research will help in CEO retention within Australian and international hospitals. Increasing turnover of CEOs has detrimental consequences toward health care organizations therefore this study will help toward better and efficient functioning of hospitals.
**What are your research’s implications toward theory, practice, or policy?**
This research has explored the qualities a successful CEO should possess, the challenges they may face in their role. Moreover, causes and consequences of increasing CEO turnover have been discussed. Most importantly, strategies for CEO retention have been recommended. The above findings will immensely help not only CEOs to perform their role better but also assist hospitals in retaining their CEOs which can lead to optimal functioning of hospitals.

## Introduction

In Australia, health executive’s turnover has been identified as a significant issue as it leads to the high financial costs, loss of human capital and adverse effects on staff work and patient care.^1^ About 25% to 50% of Chief Executive Officer (CEO) resignations within Australian hospitals are reported to be involuntary.^2^ Interestingly, the rate of CEO turnover in Australia is significantly higher than in both the US and in Europe.^1^ Studies indicate that 25% of hospitals spent between 6 months and a year to replace their CEO, and another 5% spent over a year.^1^ Similarly, in the US health care system, CEO turnover has been a critical issue with one report indicating the maximum tenure of a CEO to be 4 years.^3^ Moreover, it was found that the cost of replacing a hospital CEO will typically be around US$500,000.^4^

Health care CEOs, like many other industries, are under huge pressure by losing their jobs.^5^ The distinction in health care is that the business is going through a change in paradigm in terms of the way it does business.^6^ A major shift in the Australian conventional health care system as of now is not on getting people out of hospital, but rather on preventing them from entering it in the first place.^7^ The COVID-19 pandemic has magnified the issue of a global scarcity of healthcare professionals while risking their own health on the front lines of the pandemic resulting in additional challenges for health care leaders.^5^ The challenges a CEO faces include capital and resource management, fundraising, uncertain future, competition, complying with an overabundance of federal, state, local, and industry regulatory policies.^8^ Apart from the above, the CEO must deal with day-to-day operational issues and to ensure that the organization meets its own mission while providing a means of sustaining its future.

CEO turnover in health care has been extensively studied since late 90s as it was found to be directly related to hospitals stability. Negative consequences of increasing CEO turnover such as loss of staff, adverse patient care, executive turnover and budgetary issues have been reported.^1^ A study conducted in Australia has reported the negative impact of CEO turnover toward the hospital’s quality management. High CEO turnover adversely affected the compliance with hospitals quality assurance system, that is, to plan, monitor and improve quality of care. Staff identified a lack of vision and shifting priorities with the high CEO turnover rate, which they described as confusing and demotivating.^9^ Other studies included chief nursing officer, pharmacy director, chief finance officer as opposed to the CEO, found that the CEO turnover rate was higher compared to the other executives.^10^ In addition, the departure of the CEO is often followed by the departure of other key executives, leading to further human capital being lost in the organisation.^11^

Given the negative consequences of CEO turnover, researchers have endeavored to investigate the causes of CEO turnover. A large part of the research on CEO turnover has stressed either on inspirational or social credits of the CEO, for example, desire for a superior position somewhere else or a conflict with the board or clinical staff, that demonstrated diminished job satisfaction.^12^ Although the issue of increasing CEO turnover has been studied in the USA within different settings,^13^ up to our knowledge no studies have been conducted so far in Australia that have identified the causes for increasing CEO turnover in hospitals. Therefore, the aim of this study is to identify the qualities of CEOs, the challenges they face in the CEO role, causes and consequences of increasing CEO turnover and their retention strategies in Australia using a qualitative study design.

## Method

The study gained ethics approval from the Ethics Committee. Informed consent was obtained from all participants. The procedures followed were in accordance with the National Health and Medical Research Council of Australia National Statement on Ethical Conduct in Human research. We conducted a qualitative study using semi-structured interviews with CEOs of health care organizations.

The researcher purposefully selected participants who were working as CEOs in their current role within different states of Australia. Both public and private hospitals across Australia were chosen excluding aged care facilities. It has been confirmed that this type of selection enhances sample coverage and provides the researcher a framework for appropriate analysis.^14^ Researcher designed the interview questions (Table 1) guided by published systematic reviews^3,10^ that discussed qualities of CEO, challenges of CEO role, causes and consequences of CEO turnover in health care settings.^15,16^ During the interviews, field notes were taken, and the interviews were audio recorded so they were rechecked for accuracy for analysis.

**Table 1.** Interview Questions.

Q 1	Being a CEO is a demanding role, what qualities helped you to perform this role?
Q 2	Did you work as a CEO in your last role? If yes, please mention the reasons behind your departure? If no, then what do you see as the main challenges in your current role as CEO?
Q 3	In your opinion, what could be the possible reasons for CEO’s departure from a health care organization?
Q 4	What do you think could be the potential consequences of high CEO turnover toward health care organizations?
Q 5	Do you think board or similar governing body could support CEO’s better? Please comment on how CEO’s can be better supported.

### Procedures

The date and time of the interview were confirmed by the CEO or their assistant. Due to current COVID-19 restrictions, interviews were conducted online via MS Teams or Zoom which lasted between 15 and 60 min. Interviews followed a semi-structured schedule. Interview questions were designed to prompt discussion of CEO’s perspective on the causes and consequences for CEO turnover, the support they get from board and in general to undertake the role efficiently, their own qualities and the challenges they face within their role. These questions facilitated discussion of the participants’ perceptions regarding the causes and consequences of CEO turnover. Moreover, CEOs expressed their thoughts on how they can be supported and the role of board in supporting them. The interviews were conducted until saturation of data was achieved. They were recorded and transcribed verbatim.

### Data Analysis

Data collection and the inductive approach were subsequent until reaching the saturation of themes. Upon the completion of data collection, thematic analysis was initiated using an inductive approach until theoretical saturation was achieved.

For consistency and completeness, 2 researchers actively coded the data and identified the initial codes and themes. Researchers did the coding together and compared the codes as they worked through the transcribed text. We repeatedly read the interview data to identify new codes and themes. Each line was read carefully and systematically so the important feature and pattern could be coded. Codes were arranged into coherent categories; this was a recurrent process that was continued until all codes and themes were sorted.^17^ The identified themes and codes were repeatedly checked against the data to make sure they accurately represent the data corpus. Any discrepancies were discussed among the researchers and the overarching themes were systematically conceptualized. Connections and meaning were recognized between and within themes and subthemes.

## Results

### Participants Characteristics

Fourteen CEOs from different health care organizations agreed to be interviewed for this study. The characteristics of participants are shown in Table 2. They were 5 males and 9 females and aged from 38 to 64 years. As a CEO, they head for the executive team who oversees the directors and managers that manage the day-to-day operations of the organization, its people and resources.

**Table 2.** Participants Characteristics.

Parameter	Average/range
Age	51 (38-64 years)
Gender	5 males and 9 females
Years of experience in current CEO role	2-4
Years of experience in previous organization	2-3
Currently managing public hospital	7
Currently managing Private hospital	7
Working in Metro area	8
Working in Rural area	6
Qualifications (Master of Health Administration)	10

Ten out of 14 participants had a master’s in health management or similar qualification with some having a bachelor’s degree. Interestingly, the majority of them started their career as a registered nurse. The participants were in their current CEO role ranging from 1.8 to 30 years. All of them managed a large complex health care organization and they reported to a board of directors or group CEO’s.

### Themes Emerging From the Data

Four to Five sub-themes were identified relating to each of the 5 themes: qualities of CEOs, challenges of CEO roles, causes of CEO turnover, consequences of CEO turnover, CEO retention strategies (Table 3).

**Table 3.** Data Findings – Themes & Sub-Themes.

Themes	Sub-themes	Interview quotes
Qualities of CEOs	• Leadership qualities • Relationship management • Delegation and prioritization skills • Character/Personal traits • Industry experience • Resilience	*“You must have a strong belief that you can make a difference.” (Female Metro CEO, 61* *years)* *“You have to have I think, leadership qualities that are about bringing others up. I think you need to have a really clear sense of principles of good corporate governance.”(Female Rural CEO, 55* *years)* *“. . .. . .that it’s a lot of your business is actually outside of the day to day running of a hospital or health service. It’s relationship management quite broadly across the community.” (Male Rural CEO, 55* *years)* “*So, a combination of industry experience, particularly as a clinician so my clinical background has been an advantage. . ..”(Female Metro CEO, 58* *years)* *“You know, it literally some things just do fall off. So it is having some good support rather than just developing a good priority and having normal things, having good delegation skills. So the prioritization .”(Male Metro CEO, 57* *years)* *“I don’t believe you can be resilient in a role unless you have a real passion and belief and care deeply about what you do matters”(Female Metro CEO, 61* *years)*
Challenges of CEO role	• High expectation from the role • COVID-19 • Managing organizational change • Staff management	*“Too Many competing demands, capturing opportunities, prioritization of time, people management, human management aspects of the role always a challenging one and then how do you sell change” (Female Metro CEO, 51* *years)* *“And then I guess the second challenge has been the pandemic we have been relentlessly busy. . .”(Female Rural CEO, 55* *years)* *“The transition at the at the board level has changed. significantly. And managing that change has been really challenging. . …”(Female Metro CEO, 38* *years)* *“. . ..And then the part of that I call the seesaw is how do you manage the demand? How do you manage the workforce to then deliver that demand? And I think at the moment is a healthy balance between volume of workforce and skill, the workforce across Australia at the moment we haven’t got that?”(Male Metro CEO, 52* *years)*
Causes of CEO turnover	• Organizational • Performance • Pressure • Personal • Health reform	*“I think that unfortunately, the CEO is too easily blamed.” (Female Rural CEO, 55* *years)* *Performance is one. Like meeting the targets key performance indicators of the hospital. (Female Metro CEO, 51* *years).* *The current political environment can be difficult and so that’s another reason sometimes CEOs either appear to move on but in fact, they have been moved on (Female Metro CEO 58* *years)“And I’m sure you speak to many of them probably work 20 h a day. . .. . .”(Male Metro CEO, 52* *years)* *“I look at the personal stuff. I think there’s the work life balance issue will come for many CEOs.”(Male Metro CEO, 64* *years)* *“I find the hardest thing is to get people to think differently to be prepared to take risks potentially in the system. . .. . .. the culture of resistance and the resistance does not need to be overheard”(Female Metro CEO, 61* *years)*
Consequences of CEO turnover	• Organizational instability • Leadership instability • Staff distrust • Affecting culture • Impacts care provision	*“I think the first consequence is that it has a direct impact that instability in leadership that has a direct impact into the quality of care providers and patient experience”(Male Rural CEO, 60* *years)* *“Performance of the organization will decline. You will lose a lot of good staff.” (Female Metro CEO, 51* *years)* *“I think it really contributes to a lack of trust in the system, and the government.”(Female Rural, 55* *years)* *“I think all that summed up in this a lot of loss of corporate knowledge. It does affect the culture of the organization, because they think you know, what’s wrong with us? That we can’t keep a CEO.”(Male Metro CEO, 64* *years)* *“I guess it opens up the opportunity for skill deficiencies. . ..” (Male Rural CEO, 55* *years)*
CEO retention strategies	• Support from Professional Boards • Good IPR between Board chair and CEO • Coaching/Mentorship • Autonomy in decision making and prioritization • Appreciation/Incentives	*“So boards create that system of safety, that assurance that might be unseen to a lot of people in the organization. But in fact, it’s a true enabler.”(Female Metro CEO, 61* *years)* *“I think otherwise is to honor the delegation of authority. ”(Male Metro CEO, 57* *years)* *“Having personal regard is important between board chair and CEO, absolutely. There has to be some sort of mutual respect between the two”(Male Metro CEO, 64* *years)* *“The other bit of support that I would always recommend to any leader is to make sure that you have somebody that you can talk to about what you do, whether that’s a coach and mentor.” (Female Metro CEO, 61* *years)* *“. . …they need to invest in culture and leadership programs. . ..”(Female Rural CEO, 55* *years)* *“. . .Will be good if we get appreciated. . .”(Female Rural CEO, 53* *years)*
Abbreviations	CEO- Chief executive officer IPR- Inter personal relationship COVID-19- Coronavirus Disease of 2019	

#### Qualities of CEOs

Participants were asked about the qualities that enabled them to manage their roles. Most of the CEOs expressed that their leadership style and their professionalism helped them to perform their role. CEOs cited being resilient and having good interpersonal skills enormously helped them to undertake their role. Having a good clinical background, good time management, delegation skills and industry experience has also been identified as some of the essential qualities. One of the respondents stated that being resilient is not sufficient to do a good job, real passion and belief are essential to make a difference.

“*The most important qualities from my perspective, is actually knowing a lot about yourself. So, it’s actually having really strong self-awareness and knowing how you show up and knowing how you show up under pressure, and being aware of what how you’re like what your leadership style is, and the impact of that on others. We always have a great intent, but we sometimes don’t have the desired impact. So self-awareness is really important. The second thing is a real understanding of culture and leadership.”* (Female Rural CEO, 55 years)

#### Challenges of CEO’s role

CEOs expressed that they face so many challenges in their role which make their job onerous in health care. Most of the CEOs agreed that COVID-19 had added another level of complexity and made their job more laborious. Most of them believed that managing a budget is one of the challenging aspects of their job given the constant demand of health services with the limited availability of resources. Some rural CEOs highlighted that staff recruitment and retention have been a real dilemma and finding the right skill mix in a rural setting is crucial for efficient functioning of their health services. Finding time to do things is another most common challenge that was mentioned during the interview.

*“What happens is as a CEO in these roles, we are expected to be across everything 100% of the time, and we are expected to know everything in every detail. And that creates huge pressure on people. So that is extremely extracting.” (Female Rural CEO, 55* *years)*

*“The pressures are absolutely enormous. What we’re seeing a trend now is certainly COVID-19 related over the last 18 months or so. And so, what we’re seeing now is because we’re having to continually adapt and adopt to our internal and external environments, the neuroplasticity in our brains can be stretched in turn so much the stress that pressure is phenomenal” (Male Metro CEO 52* *years)*

#### Causes of CEO turnover

There were multiple reasons that CEOs highlighted that may contribute to their departure from a health care organization. The most common reasons mentioned were burnout, work pressure and the political environment that they must deal with. Moreover, they mentioned that the work pressure has been immense, and they are expected to know every detail about everything within their organization. Some work 18 to 20 h a day which is overwhelming and the political environment can be tricky and exhausting to manage. Another common reason that was identified during the interview was the CEO’s having issues with the board chair. If the CEO and board chair doesn’t work together, then generally it’s the CEO who leaves. Therefore, it is important to have good inter-personal relationship, trust and respect between CEO and board chair. One of the other highlighted reasons was that they got lots of scrutiny and blame as they are the face of that organization.

*“From what I would say is burnout. Especially over the last two years, when well not only Australia, the world’s been grappling with this this pandemic. You know, at the moment I see health generically is stretched. We’ve got a lot of very tired staff. We’ve got large numbers of vacancies. just that constant. Day to day pressure. Yeah, particularly in country areas. And you’re always accountable so that it’s just that constant churn, that constant availability to local communities. That just gets people down. So I think it’s the Pressure of managing a large complex system.” (Male Rural CEO, 55* *years)*

*“You know, when you talk about what will be one of the reasons for a CEO leaving, that would certainly be that breakdown in relationship between the board chair and the CEO, you couldn’t function if it wasn’t strong, open and honest. You could not function.” (Male Rural CEO 60* *years)*

#### Consequences of CEO turnover

Most of the CEO’s believe that increasing turnover of CEOs can bring negative consequences toward the organization. It can create self-doubt among staff and bring down staff trust toward the organization. This can in turn impact the culture of the organization, leading staff resistance to change, budgetary issues, leadership instability, losing management control and overall affecting the moral integrity of the organization. One of the CEOs during their interview mentioned that when a CEO leaves, it disrupts the organization for a 2-year period and frequent turnover may put the organization at risk.

*“So I think leadership instability is a risk for an organization because of the different approaches of leaders. So I think sometimes leaders can send an organization in a particular direction. So, unless there’s a strong board or strategy or executive team, then I think that that creates a potential change in direction of the organization. The change in style creates uncertainty and lack of confidence in amongst the workforce. So, I think the lead CEOs is the leader is very visible presence in the organization can provide the consistency, people feel comfortable with the consistency of a leader who’s got established relationship, but I think CEOs don’t want to stay for a long time because it can cause problems sometimes if they stay too long. So you need some turnover. But high turnover creates uncertainty and risk.” (Female metro CEO, 58* *years)*

### Recommendations for CEO Retention

All the CEO’s who were interviewed wanted the Board as their reporting line and they felt much supported having it in place. They felt that the board chair plays a crucial role in backing up CEO as required. They emphasized upon the relationship between Board chair and CEO. Both individuals should have good inter-personal relationship with each other which is built upon trust and respect. Apart from that, the importance of having a coach or mentor for a CEO and professional development opportunities was highlighted (Table 3).

*“So I think the role of the board chair and the CEO is obviously critical, and ensuring that boards have a clear role of their governance responsibility and that they don’t stray into operations so it’s very important for boards to keep their distance, setting the strategy and holding the CEO to account is very important. In the public sector, I think the board can play a role in managing some of the politics, particularly with ministers and governments where the CEO doesn’t have that power so chairs can sometimes if they have the appropriate authority, can manage some of that politics. I think that’s essentially what their role is, is to intervene at a political level. So I think that can be important. So those are the two things understanding their role and managing the politics.” (Female metro CEO, 58* *years)*

## Discussion

This is the first study to explore CEO turnover in Australia using a qualitative approach and it has been highlighted the increasing CEO turnover as a major issue for Australian and international health care organizations.^1,18^ It has been reported that, there were 41 executives occupying 18 different positions, highlighting the scope of this issue in Australia.^1^ Moreover, it has been noted that the CEO turnover rates have augmented from 14% in 2008 to 18% in 2017.^19^ This study is crucial to optimize the functioning of Australian hospitals as increasing CEO turnover can cause instability in leadership, affects the hospital’s budget and its overall function.^1^

Qualities that CEOs possess play an integral part in their success in the role. Personal attributes have been a major discussion point during the interview. To be able to manage time effectively means to be quick in decision making and executing the work in a timely manner.^20^ Similarly, in this study time management has been identified as one of the challenges in their role hence spending a lot of time in a comprehensive consultation to decide is difficult. Certain work remains pending until a CEO decides on either approving or declining the request therefore, they must be quick in making decisions.^21^ Moreover, having courage in taking risks along with being adaptable to change has been identified as important qualities. Regardless of the setting, health or non-health, a CEO needs to have qualities such as high intelligence, high self-efficacy, and high self-monitoring. High level of general intelligence should help a CEO in simplifying complex competitive situations. Higher self- efficacy is likely to inspire and motivate staff and high self- monitoring is likely to aid CEOs to communicate with distant stakeholder groups.^22^

CEO role brings a certain number of challenges which were clearly identified during the interview process. The biggest challenges hospital CEOs are facing in 2022 is attracting, hiring, retaining, developing, growing, and engaging talent^23^ which are aligned with our study findings. These challenges were notable during the pandemic. Coronavirus disease 2019 (COVID-19) is stretching the capacity of the health system and it impacted hospital finances in multiple ways such as through increased uncompensated care, given the rise in insurance associated with the surge in unemployment caused by the COVID-19, and through revenue losses due to cancelations or postponements of elective outpatient procedures, which specifically rural hospitals rely on for their revenue.^24^ Literature suggests that rural hospital experienced frequent CEO turnovers compared to metro counterparts due to limited resources^12^and it was expressed that COVID -19 has increased resource constraints specifically in rural hospitals. Moreover, the pandemic had a severe impact on clinicians work environment, which resulted in burnout and staff turnover.^25^ We found in this study that the competing demands of the financial sustainability of the business and balancing it with patient safety and service delivery are the main challenge which is no different in either public or private setting.

Some CEOs are not willing to disclose the actual nature of their termination to the researchers,^3^ therefore the real number of forced resignations is expected to be higher than reported. Interestingly, a CEO stated, it appears that CEOs move on, but in fact in many cases they have been asked to move on. Interviews highlighted that CEO gets easily blamed for not achieving the targets and spending over the allocated budget. It creates an enormous pressure for the CEO to find balance between service provision and finance while managing government politics.^16^ Further, a CEO gets personally, ethically, and morally challenged when they are asked to make changes from a ministerial level that are fundamentally against their core principles hence, they may decide to leave. A public organization CEO faces more politics as compared to a private organization CEO therefore the previous research has revealed that turnover was found to be less in private sector compared to public^26^ Even though, private hospitals are more financially focused, but they give their CEOs higher degree of autonomy and tools to manage their organization which is lacking in public hospitals. Private hospital CEOs do not necessarily have to manage to the political influences, but by the same token, they have a similar sort of pressure from their private health insurers to their board. Unsupportive board adds further complexities to a CEOs role therefore a strong emphasis was made on a good interpersonal relationship between a CEO and board chair. Expectancy theory reveals that individuals are motivated to perform work related acts in the anticipation of getting desired outcomes or rewards. This theory suggests that individual must both desire the preferred anticipated outcome and have the expectation that the outcome will be obtained.^27^ To apply the expectancy theory to the study of CEO turnover, we must determine what their expectations and preferences are. CEO interviews clearly indicated that they are working under high pressure environment, and it is challenging to get the desired results. They do expect to deliver better results for their hospitals and to get recognized and rewarded for it however it was expressed that they get easily scrutinized and blamed for any issues and there is lack of reward in the system. When they see a lack of appreciation for their work, it demotivates them to stay further in the role resulting in them resigning or boards terminating them for not achieving targets.

Consequences of frequent CEO turnover proved detrimental to organizational stability and was linked to massive cost to the organization. Due to the revolving door of CEOs, it is very hard to achieve a fundamental change in the system because the people directing it keeps on changing. A crucial event in an organization that affects performance and organizational processes is the departure (or succession) of the CEO.^28^ It takes 5 years for a CEO to make a positive change and reinforce those changes on an organization however if CEO leaves before the 5-year period, then the organization may revert to its former self.^29^ From the CEOs point of view, if they stay in a role for 5 years then it’s beneficial for the organization as the changes will be successfully embedded in the hospital, however, CEOs should not stay for a long time because it can cause problems and organization may need a fresh set of eyes.^4^ Moreover, from a CEOs career perspective, it’s a reasonable time to stay in one job. CEOs as the leader must make their presence visible in the organization which can provide the consistency and people feel comfortable with the consistency of a leader who’s got established relationship. CEOs can send an organization in a particular direction so unless there is a strong board or strategy or executive team, it can create a potential change in direction of the organization. The change in style creates uncertainty and lack of confidence in amongst the workforces.^9^

It is a fine balance to have good interpersonal relation between the board chair and CEO, because sometimes chair is advocating on behalf of the organization, but also sometimes the chair is holding the CEO to account. There is a level at which they need to maintain the level of separation between the chair and not become too involved, and that really occurs when they have a professional chair who understands the role of the board in terms of governance.^30^ There are measures that may reduce CEO turnover such as communication between CEOs and their boards, including “measurable expectations” for the CEO with regular progress reviews. If hospitals take these steps, there’s going to be less CEO turnover within the hospitals.^28^ Boards need to advocate and invest in culture and leadership programs. In addition, it is important to recruiting the right people with the right leadership skills in executive roles. In both private and public hospitals, the strategy, direction, and the approach should be unified and agreed upon by the board. Moreover, in either setting the board members should be providing advice and guiding CEOs in their roles. Apart from that, it was highlighted during the interview that boards play a vital role in managing politics particularly with ministers and governments where the CEOs does not have that power. Board chairs can manage the politics if they have appropriate authority, and it is essentially their role to intervene at a political level.

This study indicates that the CEO turnover issue is evident in Australian hospitals, however it can be minimized if CEOs are properly supported by the board. The board needs to ensure that their goals and visions are aligned with each other, and they need to work with the CEO to achieve them. They need to give their CEO autonomy in decision making and it is critical for government officials to involve CEOs when making crucial decisions that may affect their organization. Importantly, CEO should be supported for their own professional development to learn new skills and knowledge to apply in their role.

## Study Limitation

The findings of this study may be limited as the sample was confined to 14 CEO’s working across Australia. Further, calculation of sample size and power analysis was not performed for this study which may influence the results. CEOs from both public and private, rural, and metro hospitals were interviewed therefore the settings and challenges included in this study may vary across settings. With online interviewing the body language which may signify certain emotions may not be captured affecting the robustness of the data captured.

## Implications for Practice and Research

Increasing CEOs turnover on a frequent basis can be detrimental to health services therefore this study highlighted certain strategies to minimize turnover, however further studies can help to identify the benefits of some of these strategies in addressing this issue. Exploring the causes and strategies for CEO retention will be extremely important for better functioning of hospitals.

## Conclusion

Our study identified several qualities of CEOs, the challenges they face in their role, the reasons behind their departure, the consequences of their turnover and support they require to do their role well. CEOs expressed that their roles are very demanding, and they require significant time which may lead to burnout. CEOs may leave the organization due to the organizational, performance, and personal factors. Frequent turnover causes leadership instability and may alter organizational stability. Therefore, this study indicated that CEOs would benefit from appropriate support of the board and its chairs to perform their role optimally.
